# Comparative proteomic analysis of *Lactobacillus plantarum *for the identification of key proteins in bile tolerance

**DOI:** 10.1186/1471-2180-11-63

**Published:** 2011-03-29

**Authors:** Erwann Hamon, Peter Horvatovich, Esther Izquierdo, Françoise Bringel, Eric Marchioni, Dalal Aoudé-Werner, Saïd Ennahar

**Affiliations:** 1 Equipe de Chimie Analytique des Molécules Bio-Actives, IPHC-DSA, Université de Strasbourg, CNRS, 67400, Illkirch, France; 2 Aérial, Parc d'Innovation, Illkirch-Graffenstaden, France; 3 Department of Analytical Biochemistry, Centre for Pharmacy, University of Groningen, Groningen, The Netherlands; 4 Laboratoire de Génétique Moléculaire, Génomique, Microbiologie, Université de Strasbourg, CNRS, 67083, Strasbourg, France

## Abstract

**Background:**

Lactic acid bacteria are commonly marketed as probiotics based on their putative or proven health-promoting effects. These effects are known to be strain specific but the underlying molecular mechanisms remain poorly understood. Therefore, unravelling the determinants behind probiotic features is of particular interest since it would help select strains that stand the best chance of success in clinical trials. Bile tolerance is one of the most crucial properties as it determines the ability of bacteria to survive in the small intestine, and consequently their capacity to play their functional role as probiotics. In this context, the objective of this study was to investigate the natural protein diversity within the *Lactobacillus plantarum *species with relation to bile tolerance, using comparative proteomics.

**Results:**

Bile tolerance properties of nine *L. plantarum *strains were studied *in vitro*. Three of them presenting different bile tolerance levels were selected for comparative proteomic analysis: *L. plantarum *299 V (resistant), *L. plantarum *LC 804 (intermediate) and *L. plantarum *LC 56 (sensitive). Qualitative and quantitative differences in proteomes were analyzed using two-dimensional electrophoresis (2-DE), tryptic digestion, liquid chromatography-mass spectrometry analysis and database search for protein identification. Among the proteins correlated with differences in the 2-DE patterns of the bacterial strains, 15 have previously been reported to be involved in bile tolerance processes. The effect of a bile exposure on these patterns was investigated, which led to the identification of six proteins that may be key in the bile salt response and adaptation in *L. plantarum*: two glutathione reductases involved in protection against oxidative injury caused by bile salts, a cyclopropane-fatty-acyl-phospholipid synthase implicated in maintenance of cell envelope integrity, a bile salt hydrolase, an ABC transporter and a F0F1-ATP synthase which participate in the active removal of bile-related stress factors.

**Conclusions:**

These results showed that comparative proteomic analysis can help understand the differential bacterial properties of lactobacilli. In the field of probiotic studies, characteristic proteomic profiles can be identified for individual properties that may serve as bacterial biomarkers for the preliminary selection of strains with the best probiotic potential.

## Background

Research efforts are currently underway in order to better understand the host-microbe interactions that occur in the human gastrointestinal (GI) tract [1,2]. Evidence suggests that the upset of the GI microflora balance underlies many diseases and that therapies often start with the restoration of a healthy balance [3]. In this respect, probiotics (*i.e*. "live organisms that, when administered in adequate amounts, confer a health benefit on the host" [4]) are gaining widespread recognition as new prevention strategies or therapies for multiple GI diseases [5].

Lactic acid bacteria (LAB) are indigenous inhabitants of the human GI tract [6]. They also have a long history of traditional use in many industrial and artisanal plant, meat, and dairy fermentations. Based on their putative or proven health-promoting effects, these bacteria are commonly marketed as probiotics [7]. Some LAB strains have clearly been shown to exert beneficial health effects [8]. However, these effects are known to be strain specific [9], and the underlying molecular mechanisms remain poorly understood [10]. The level of evidence provided varies greatly depending on studies, and effects associated with most of the marketed products remain unsubstantiated. Current legislations agree to call for scientific substantiation of health claims associated with foods, mainly through well-designed human intervention clinical studies [11]. Therefore, scientific evidence that would help understand the mechanisms behind the activities of probiotics and narrow down the expensive and time-consuming clinical trials to strains that stand the best chance of success are of great interest. Such evidence may include data from epidemiological studies, from *in vivo *and *in vitro *trials, as well as from mechanistic, genomic and proteomic studies.

Proteomics plays a pivotal role in linking the genome and the transcriptome to potential biological functions. As far as probiotics are concerned, comparative proteomics can be used in the identification of proteins and proteomic patterns that may one day serve as bacterial biomarkers for probiotic features [12]. Comparison of differentially expressed proteins within the same strain in different conditions have been performed, shedding light on bacterial adaptation factors to GI tract conditions, such as bile [13-16], acidic pH [18,19], and adhesion to the gut mucosa [20,21]. On the other hand, 2-DE coupled with mass spectrometry (MS) has been used to analyze bacterial protein polymorphisms and to distinguish between closely related pathogenic organisms [22-25], but this approach has rarely been employed to compare strains based on their probiotic features. We previously reported the first study of this kind which highlighted key proteins involved in the adhesion properties of *Lactobacillus plantarum *to mucin [12]. Recently, hydrophobicity and cell agglutination properties in *Bifidobacterium longum *were investigated through the protein patterns of four strains [26]. Both studies focused on cell surface properties related to adhesion. To our knowledge, proteomics has not been used to compare intra-species strains as regards other GI tract adaptation factors.

Yet, the ability to survive exposure to bile is one of the commonly used criteria to select potential probiotic strains, since bile is a major challenge for bacteria entering the GI tract [4]. In addition to affecting membrane characteristics, bile has numerous other effects on bacterial cells including detergent action, DNA damage, acid, oxidative and osmotic stresses [27]. Thus, when it comes to the study of bile stress, the overall bile, oxidative, acid, detergent and salt (BOADS) stresses should be taken into account. Although mechanisms of survival to bile stress are not fully understood, several genes and molecules involved in this process have been indentified in lactobacilli [28].

The latter remain the most prominent group of probiotic bacteria, despite the increasing use of other genera such as bifidobacteria. Widely studied with regard to numerous properties, they represent a suitable bacterial model. Among the most common species, *L. plantarum *is part of a number of ethnic as well as commercial probiotic preparations where it has a long history of safe use [29]. In addition, it is an important member of the GI tract microbiota and is a flexible and versatile species with one of the largest genomes known within LAB [30].

The present paper investigates the natural protein diversity within the *L. plantarum *species with relation to bile tolerance and subsequent ability to resist GI tract conditions. This investigation is based on the study of the proteomic profiles of three *L. plantarum *strains selected according to their *in vitro *bile tolerance properties.

## Results

In this study, three strains showing different levels of bile tolerance ability *in vitro *were chosen out of nine *L. plantarum *subsp. *plantarum *cultures (Table 1). The selected strains were cultured in non-stressing conditions so as to investigate their inherent proteome differences, with a specific focus on proteins that may play a role in bile tolerance processes. In addition, changes in protein expression during bile salt exposure were analyzed in order to assess the effective involvement of the proteins of interest in the bile stress response of the three strains.

**Table 1:** Sources of bacterial strains

**Bacterial strain**^**a**^	Provider	Origin
LC 56	Aerial^b^	Corn silage
LC 660	Aerial^b^	Grass silage
WHE 92	Aerial^b^	Munster cheese
LC 800	Aerial^b^	Horseradish
LC 804	Aerial^b^	Olives
CECT 748^T^	CECT^c^	Pickled cabbage
CECT 749	CECT^c^	Pickled cabbage
CECT 4185	CECT^c^	Silage of vegetable matter
299 V	Probi^d^	Human intestinal mucosa

a) Identification based on PCR amplification targeting the *recA *gene [51].

b) Aerial, Illkirch, France.

c) Spanish Type Culture Collection, Valencia, Spain.

d) Probi, Lund, Sweden.

### Bile salt tolerance

*L. plantarum *strains were exposed to bile stress using increasing Oxgall concentrations. The effects of 0.5%, 1.0%, 1.8% and 3.6% Oxgall (w/v) on the maximum growth rates were investigated (Table 2). Two-way analysis of variance (ANOVA) revealed significant effects of both the bile concentration and the strain (p < 0.05). A stepwise increase in the Oxgall concentration resulted in a gradual decrease in the maximal growth rate for all strains except *L. plantarum *CECT 748^T ^and CECT 749 (p < 0.05). Strains could be assigned to three groups according to their bile sensitivity. *L. plantarum *299 V and LC 660 showed the best ability to grow in Oxgall-supplemented culture broth with relative growth rates that ranged from 85.5 ± 3.0 to 97.1 ± 1.4%, as compared to standard conditions. *L. plantarum *LC 56 was the most sensitive strain to bile salts, with relative growth rates from 19.9 ± 3.7 to 58.2 ± 0.5%. The six other strains tested were moderately bile tolerant and had relative growth rates in the range of 66.8 ± 2.5 to 81.7 ± 1.0%. *L. plantarum *LC 56 (highest decrease in growth rate), *L. plantarum *LC 804 (intermediate decrease in growth rate) and *L. plantarum *299 V (smallest decrease in growth rate) were used for comparative proteomic analysis in standard conditions and following bile salt exposure.

**Table 2:** Effect of bovine bile concentration on the relative growth rates of *L. plantarum *strains

Strains	Relative growth rate* (% μ) with Oxgall concentrations (% [w/v])				
	
	Control	0.5	1.0	1.8	3.6
299 V	100	97.1 ± 1.4^a^	96.3 ± 1.2^a^	93.5 ± 2.9^a^	91.2 ± 2.3^a^
LC 660	100	93.9 ± 0.8^a^	94.2 ± 2.0^a^	89.6 ± 1.7^a^	85.5 ± 3.0^b^
CECT 748	100	81.7 ± 1.0^b^	80.3 ± 0.6^b^	80.5 ± 1.8^b^	79.1 ± 0.9^c^
CECT 4185	100	78.5 ± 2.2^b,c^	78.3 ± 0.7^b,c^	74.5 ± 2.6^c^	71.6 ± 2.1^d^
WHE 92	100	79.1 ± 2.4^b,c^	76.2 ± 1.1^c^	72.3 ± 4.3^c^	66.9 ± 0.5^d,e^
LC 804	100	76.2 ± 1.7^c,d^	76.6 ± 0.9^c^	72.8 ± 1.3^c^	68.4 ± 1.5^e^
LC 800	100	74.1 ± 3.6^d^	67.9 ± 1.6^d^	66.3 ± 2.0^d^	66.5 ± 1.6^e^
CECT 749	100	69.6 ± 1.9^e^	68.9 ± 3.2^d^	68.1 ± 1.4^d^	66.8 ± 2.4^e^
LC 56	100	58.2 ± 0.5^f^	45.5 ± 2.5^e^	39.4 ± 1.4^e^	19.9 ± 3.7^f^

*Data are expressed as a percentage of the growth rate (h^-1^) obtained in the absence of bile, which was assigned a value of 100%. Means ± standard deviations of three independent experiments with three replicates per assay are given. Means in the same column with different letters (a through f) differ (p < 0.05).

### Comparative proteomic analysis of *L. plantarum *strains in standard growth conditions

**Figure 1:**
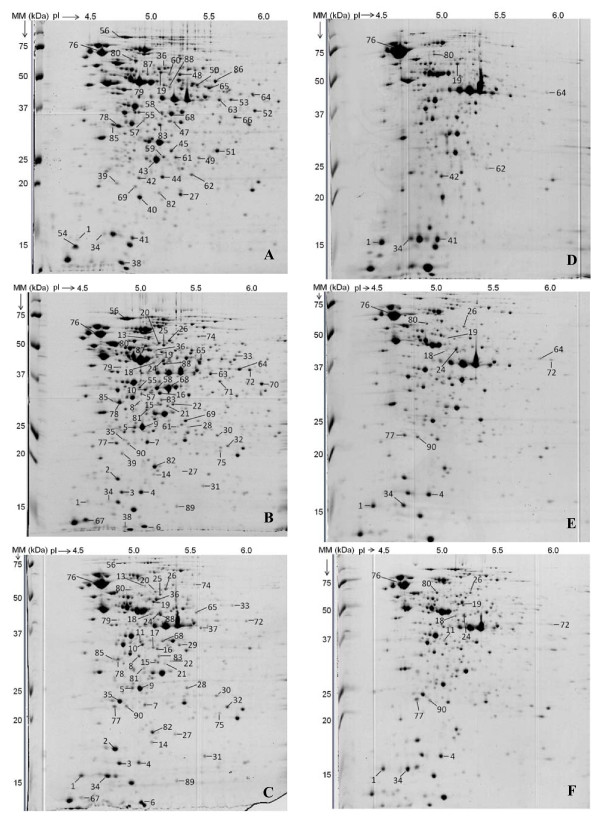
**2-DE gels of whole cell proteomes from *L. plantarum *LC 56, LC 804 and 299 V cultured in standard and bile-stressing conditions**. The figure shows representative 2-DE gel pictures (pH range: 4-7) of whole-cell protein lysates from early stationary phase of *L. plantarum *LC 56 (A and D), LC 804 (B and E), and 299 V (C and F) cultured without (A-C) and with (D-F) 3.6% (w/v) Oxgall. Spots exhibiting differential expression between strains in standard growth conditions and identified by LC-MS analysis are labeled (A-C), with a focus on expression changes after bile exposure for proteins previously reported as being involved in bile tolerance processes (D-F).

*L. plantarum *LC 56, LC 804 and 299 V were cultured under non-stressing conditions and cell proteins were extracted. Protein loads of 150 μg representing total proteomes of each of the three strains were separated by 2-DE. Three independent biological replicates were carried out per strain. Figure 1(A-C) shows representative 2-DE patterns for the three strains when cultured in standard conditions. Inter-strain discrepancies between inherent proteomic patterns were investigated with regard to the different bile tolerance abilities of the strains, so as to pinpoint proteins that may be implicated in the bile tolerance process.

Although the overall inherent protein patterns of the three *L. plantarum *strains were similar, 90 out of an average of 400 detected protein spots displayed different abundance levels in standard conditions (Additional file 1). The corresponding gel spots were excised and subjected to tryptic digestion followed by liquid chromatography-mass spectrometry (LC-MS) analysis and proteomic database search using Phenyx and OMSSA to elucidate their identity and likely function. Proteins in a total of 80 spots were identified, some of which were found in more than one spot, indicating the presence of protein isoforms. Proteins fell into 13 functional categories, covering most of the biochemical functions encountered in bacterial cells. Sequence alignment analysis focused on the three sequenced *L. plantarum *strains WCFS1, JDM1 and ATCC 14917 revealed a systematic occurrence of the corresponding genes with high levels of similarity (> 98%, results not shown).

Among the proteins with differential abundance levels between strains that were identified in non-stressing conditions, 15 have previously been reported to be involved in BOADS stress tolerance processes (Table 3): (i) five proteins (α-small heat shock protein 1 (Hsp1), spot 1; bile salt hydrolase 1 (Bsh1), spot 11; glucose-6-phosphate 1-dehydrogenase (Gpd), spot 26; GroEL chaperonin (GroEL), spot 76; F0F1 ATP synthase subunit δ (AtpH), spot 90) were exclusively detected or significantly more abundant (p < 0.05) in the resistant strain (299 V); (ii) three proteins (glycine/betaine/carnitine/choline ABC transporter (OpuA), spot 18; glutathione reductase 1 (GshR1), spot 24; and ATP-dependent Clp protease proteolytic subunit, spot 77) were present at the same level in both resistant and intermediate strains (299 V and LC 804), but not observed in the sensitive strain (LC 56); (iii) two proteins (α-small heat shock protein 3 (Hsp3), spot 4; and bifunctional GMP synthase (GuaA), spot 80) were present solely or to a higher extent in the intermediate strain; (iv) one protein (glutathione reductase 4 (GshR4), spot 19) showed the same expression level in the resistant and sensitive strains, while it was barely detected in the intermediate strain; (v) two proteins (stress-induced DNA binding protein (Dps), spots 34 and 41; cyclopropane-fatty-acyl-phospholipid synthase (Cfa2), spots 64 and 72) displayed different expression levels between strains depending on the considered isoform; and (vi) two proteins (dTDP-4-dehydrorhamnose 3,5-epimerase (RfbC), spot 42; and ribosomal protein S30EA, spot 62) were only detected in the sensitive strain. These 15 proteins belonged to 8 functional categories, including cell membrane biogenesis, molecular transport, energy metabolism, as well as chaperone activity.

**Table 3:** Impact of a 3.6%-Oxgall exposure on specific proteomic patterns putatively related to bile tolerance

Functional category	Protein	**Stress**^**a)**^	**Gene**^**b)**^	Spot number	**Normalized volume with 3.6% Oxgall**^**c)**^			**Variation factor: bile *vs*. standard conditions**^**d)**^		
						
					LC 56	LC 804	299 V	LC 56	LC 804	299 V
Translation, ribosomal structure and biogenesis	Ribosomal protein S30EA	B [14]	lp_0737	62	0.049 ± 0.004	-	-	-3.2	-	-
Posttranslational modification, protein turnover, chaperones	α-Small heat shock protein	O [55]	lp_0129 (*hsp1*)	1	0.952 ± 0.059	1.008 ± 0.190	0.597 ± 0.082	34	11.4	2.1
			lp_3352 (*hsp3*)	4	-	1.172 ± 0.159	0.744 ± 0.171	-	1.7	2.2
	Chaperonin GroEL	B [14]	lp_0728 (*groEL*)	76	27.427 ± 1.216	14.137 ± 0.142	11.931 ± 0.715	3.7	1.9	-1.1*
	ATP-dependent Clp protease	D [56]	lp_0786 (*clpP*)	77	-	0.360 ± 0.072	0.282 ± 0.020	-	2.0	1.7
Energy production and conversion	F0F1 ATP synthase subunit delta	B [44]	lp_2367	90	-	0.243 ± 0.051	0.110 ± 0.012	-	4.3	1.2*
	Glutathione reductase	O [57]	lp_3267 (*gshR4*)	19	0.179 ± 0.023	0.011 ± 0.001	0.210 ± 0.008	-1.8	-1.8	-1.3
			lp_0369 (*gshR1*)	24	-	0.314 ± 0.025	0.148 ± 0.009	-	1.1*	-1.6
Carbohydrate transport and metabolism	Glucose-6-phosphate 1-dehydrogenase	B [14], O [58]	lp_2681 (*gpd*)	26	-	0.098 ± 0.005	0.116 ± 0.025	-	-1.2*	-1.4
Amino-acid transport and metabolism	Glycine/betaine/carnitine/choline ABC transporter	B [48], S [58]	lp_1607 (*opuA*)	18	-	0.034 ± 0.003	0.081 ± 0.007	-	-1.6	1.5
Nucleotide transport and metabolism	Bifunctional GMP synthase/glutamine amidotransferase protein	A [35]	lp_0914 (*guaA*)	80	0.039 ± 0.003	0.104 ± 0.009	0.209 ± 0.016	-7.6	-1.8	12.5
Inorganic ion transport and metabolism	Stress-induced DNA binding protein	O [59]	lp_3128 (*dps*)	34	0.278 ± 0.026	0.074 ± 0.003	1.212 ± 0.124	2.6	2.0	1.0*
				41	0.957 ± 0.077	-	-	2.5	-	-
Cell wall/membrane/envelope biogenesis	Bile salt hydrolase	B [49]	lp_3536 (*bsh1*)	11	-	-	0.061 ± 0.008	-	-	-2.6
	dTDP-4-Dehydro-rhamnose 3,5-epimerase	O, D [60]	lp_1188 (*rfbC*)	42	0.151 ± 0.010	-	-	1.1*	-	-
	Cyclopropane-fatty-acyl-phospholipid synthase	A [42,43]	lp_3174 (*cfa2*)	64	0.0312 ± 0.002	0.069 ± 0.007	-	-6.9	-2.5	-
				72	-	0.046 ± 0.004	0.052 ± 0.001	-	-2.6	1.0*

a) Reported implication of the protein in bile (B), oxidative (O), acid (A), detergent (D) and/or salt (S) stress tolerance with the corresponding references.

b) Gene accession number in the NCBI database for *L. plantarum *WCFS1 with the general symbol of the gene in brackets.

c) Normalized relative volumes, expressed as a percentage of total valid spots. Values are means ± standard deviations; n ≥ 3 for each strain. -, not detected.

d) r = volume with bile salt/volume without bile salt for the considered strain. When r > 1, variation factor = r. When r < 1, variation factor = -1/r.

* means of volumes with and without Oxgall are not statistically different (Student's t test for paired samples, p < 0.05).

These patterns gather differentially expressed proteins in standard growth conditions among *L. plantarum *LC 56, LC 804, and 299 V that have previously been reported to be involved in BOADS stress tolerance based on dedicated mutant analysis. The impact of exposure to bile is assessed through protein expression comparison for early stationary cells cultured with and without Oxgall, using normalized relative volumes. Normalized volumes in standard conditions are listed in Additional file 1.

### Bile influence on expression levels of proteins reportedly involved in bile tolerance

Cells were cultured in stressing conditions using 3.6% Oxgall for 14 h (strain 299 V), 16 h (strain LC 804) and 20 h (strain LC 56), which allowed the harvesting of all cells at the early-stationary phase, as in non-stimulating conditions (data not shown). As protein expression is growth-phase dependent, having cells in a comparable physiological state was in fact key in this investigation. Analysis of changes in protein expression during bile salt exposure was focused on the 15 proteins previously reported to play a role in BOADS stress tolerance. Figure 1(D-F) illustrates representative 2-DE patterns for the three strains when cultured with 3.6% Oxgall. While these patterns looked similar to each other, they were quite different from those obtained in standard conditions, suggesting quantitative changes for most of the protein spots observed. Table 3 reports changes in spot intensities between standard and bile stress conditions for the 15 proteins of interest in this study. Thirteen out of the 15 proteins linked to BOADS stress tolerance in previous studies appeared to respond to the presence of bile (absolute value of fold-change factor r > 1.5, as previously described [14]), suggesting a direct involvement of these proteins in the bile tolerance process of the studied *L. plantarum *strains. These proteins could be divided into three groups. Three proteins showed higher expression levels in stressing conditions: Hsp1, spot 1 (2.1 ≤ r ≤ 34); Hsp3, spot 4 (1.7 ≤ r ≤ 2.2); and ClpP, spot 77 (1.7 ≤ r ≤ 2.0). Conversely, two other proteins were repressed when challenged with Oxgall: Bsh1, spot 11 (r = -2.6); and ribosomal protein S30EA, spot 62 (r = -3.2). The third group includes eight proteins with modifications in expression levels that depended on strains (OpuA, spot 18; GshR4, spot 19; GshR1, spot 24; GroEL, spot 76; GuaA, spot 80; and AtpH, spot 90) or resulted in a different expression of protein isoforms (Dps, spots 34 and 41; Cfa2, spots 64 and 72). The expression levels of two proteins (Gpd, spot 26; and RfbC, spot 42) however were not impacted following exposure to 3.6% Oxgall (absolute value of variation factor r ≤ 1.5), suggesting a minor role for these in the bile tolerance process of the considered *L. plantarum *strains.

## Discussion

This paper reports the application of 2-DE and MS analysis to investigate LAB proteins that are key in the bile tolerance process, a major factor when it comes to probiotics adaptation to the GI tract. Although 2-DE has known limitations and only explores part of bacterial proteomes as compared to other gel-less analyses [31], it is a widely used and affordable technique which proved to be valuable in discriminating strains according to their bacterial features [22-25]. With regard to probiotic research, two previous studies used a similar approach to explore adhesion properties of *L. plantarum *[12] and *B. longum *[26]. However, this is the first time that an attempt is made towards getting a broad picture of bile tolerance at the species level rather than focusing on a single strain.

*L. plantarum*, a versatile species with marketed probiotic strains, was chosen as a model for this study. An *in vitro *test was used to assess bile tolerance of nine strains, including *L. plantarum *299 V, a probiotic with outstanding bile resistance properties [32]. These properties were confirmed in our study, as this strain showed the best ability to grow in bile supplemented culture broths. Considerable variations in growth rates were observed between strains, with the highest effect of bile on *L. plantarum *LC 56, which is in accordance with previous reports showing a strain-specific behavior of LAB with regard to bile tolerance [33,34]. Strains LC 56 (weak bile tolerance), LC 804 (intermediate bile tolerance) and 299 V (strong bile tolerance) were selected for the proteomic investigation. For that purpose, we focused on the whole cell proteomes, since the ability of an organism to tolerate bile may require a wide array of proteins implicated in either membrane- or cytosol-based functions and mechanisms [27].

The differentially expressed proteins among the three selected strains cultured in standard conditions all appeared to be encoded by highly conserved genes in the *L. plantarum *species. These core-genome proteins are of great interest in the search for bacterial biomarkers as their relative abundance is likely to be assessed for any *L. plantarum *strain. In our case, 10 proteins displayed increasing levels of expression from the sensitive strain (LC 56) to the resistant one (299 V), suggesting a positive correlation of these proteins with bile resistance. Conversely, 4 proteins showed decreasing levels of expression as the considered strain was more tolerant to bile, indicating a link with bile sensitivity. Therefore, these proteins might represent potential biomarker candidates of bile tolerance in *L. plantarum *and should be further studied, especially the ones with unknown functions (protein of unknown function lp_2652, spot 31; putative alkaline shock proteins 1 and 2, spots 3 and 2 respectively).

Particular interest was in differentially expressed proteins with a reported putative involvement, not specifically in bile tolerance, but in the overall BOADS stress tolerance, since the deleterious effects of bile not only include a detergent action, but also low-pH, oxidative and osmotic stresses [27]. This led to the identification of 15 proteins likely to be implicated in bile tolerance of the selected strains. Two of these proteins (GuaA and ribosomal protein S30EA) have previously been negatively correlated to constitutive acid [35] and bile [14] tolerance, respectively, suggesting they could impart bacterial sensitivity to theses stress factors. Interestingly, they were not detected (ribosomal protein S30EA) or naturally underexpressed (GuaA) in the resistant strain. On the other hand, the 13 remaining proteins have been linked to BOADS stress resistance in previous studies. Ten of them were overexpressed in the resistant or intermediate strains, while only one of them displayed higher expression levels in the bile sensitive strain. These results showed that the natural protein diversity observed among *L. plantarum *strains cultured in standard conditions can reflect their ability to tolerate bile. The more resistant a strain is to bile, the more it naturally expresses proteins that can help in the bile resistance process, but also the less it produces proteins that may impart sensitivity to this stress. These proteins could therefore constitute an inherent and characteristic proteomic profile that is indicative of bile tolerance.

To confirm the putative involvement of the 15 proteins of interest in the bile tolerance process and get an overview on how bile salts affect their levels of expression, proteomic analysis of strains response to bile exposure was performed. Thirteen proteins appeared to be directly implicated in bile stress adaptation, since their expression was significantly affected by exposure to bile salt (p < 0.05). Five of them (ClpP, Dps, GroEL, Hsp1, and Hsp3) are general stress-response proteins involved in repair and protection of proteins and DNA. They were up-regulated in response to bile challenge, which is in accordance with previous findings [14,16,36-38]. This set of proteins intervenes in numerous stress-management response systems, suggesting they have unspecific contributions to bile stress tolerance, which may result in multifaceted stress-dependent mechanisms of action, as this was recently reviewed for Dps [39]. Two other proteins (GuaA and ribosomal protein S30EA) are part of regulatory systems modulating protein translation during environmental stresses. GuaA, involved in guanine nucleotide metabolism, indirectly governs intracellular GTP level responsible for translation efficiency [35], while ribosomal protein S30EA limits protein synthesis by reducing translation initiation [40]. Both proteins were down-regulated in the sensitive strain following bile exposure, which is consistent with previous studies [14,38]. All in all, 7 out of the 13 proteins directly involved in bile tolerance of the three-selected *L. plantarum *strains were not dedicated to one of the damaging effects of bile, but covered a wide range of environmental stresses instead.

In contrast, other factors contribute in a specific way to bile tolerance. This is the case of GshR1 and GshR4 which help protect the cell against oxidative injury [41]. This coincides with the lower global levels of glutathione reductases in the sensitive strain in both standard and stimulating conditions found in our study. Another protein, the Cfa2, catalyzes the cyclopropane ring formation in phospholipid biosynthesis, which may help maintain integrity of the cell envelope. In *Escherichia coli*, the cytoplasmic membrane of a *cfa*-mutant displayed increased overall permeability to protons compared to the native strain [42]. This could for instance explain the higher acid sensitivity of a *cfa*-mutant of *L. acidophilus *NCFM [43]. In our study, a Cfa2 isoform was absent in the sensitive strain, while another isoform was not detected in the resistant one, suggesting different functional properties of the isoforms with regard to bile tolerance.

Another specific mechanism of bile adaptation is the active removal of bile-related stress factors. Such is the case of the F0F1-ATP synthases which facilitate the extrusion of protons from the cytoplasm by proton motive force [28]. Previous findings reported that a bile-adapted *B. animalis *strain was able to tolerate bile by inducing proton pumping by a F0F1-ATP synthase, therefore tightly regulating the internal pH [44]. In our study, a representative F0F1-ATP synthase, AtpH, was absent in the weak strain and was up-regulated in the intermediate strain, which is consistent with the up-regulation of the corresponding gene reported for *L. plantarum *WCFS1 when exposed to porcine bile [45]. ABC transporters are also a major part of the efflux systems involved in the transport of harmful-compounds and cell detoxification [46]. A representative ABC transporter, OpuA, was more abundant in the resistant strain, less abundant in the intermediate one, and not detected in the sensitive one. This protein is known to be implied in the *L. plantarum *response to osmotic stress, one of the numerous deleterious effects of bile [47]. In addition, deletion of an *opuA *gene in *Listeria monocytogenes *was shown to significantly increase bacterial sensitivity to physiological concentrations of human bile [48]. This protein is therefore likely to be a key determinant of the high bile resistance of strain 299 V.

When it comes to bile tolerance, Bsh is probably what first comes to mind, since it involves the direct hydrolysis of bile salts. Although the ecological significance of microbial Bsh activity is not yet fully understood, the suggestion was made that it may play a major detoxification role [27]. *L. plantarum *strains carry four *bsh *genes (*bsh1 *to *bsh4*). *Bsh2*, *bsh3 *and *bsh4 *are highly conserved among *L. plantarum *species, while *bsh1 *is not and seems to be the major determinant of the global Bsh activity of *L. plantarum *strains. Besides, a *bsh1*-mutant of *L. plantarum *WCFS1 displayed a decreased tolerance to glycine-conjugated bile salts [49]. In our study, a Bsh1 homologue could only be found in the most resistant strain in standard conditions, but its amount decreased following the strain's exposure to bile. This result contrasts with the *bsh1 *gene up-regulation in *L. plantarum *WCFS1 following bile challenge [45]. Strains from *L. acidophilus *and *L. salivarius *on the other hand did not seem to up-regulate their Bsh1 production following bile exposure [38,50]. Such discrepancy in regulation trends of *bsh *genes suggests that, depending on the considered strains and species, Bsh activity may or may not be a major determinant of bile resistance.

Finally, it appeared that the six bile tolerance factors described above may contribute in various ways to the bile tolerance of *L. plantarum *strains. In particular, strains appeared to regulate key proteins differently following exposure to bile, which suggests that several strategies coexist in the bile adaptation process of *L. plantarum *species, some strains favoring certain specific pathways, while others downplaying them.

## Conclusions

This work used comparative and functional proteomics to analyze cell-free protein extracts from three *L. plantarum *strains with different bile resistance properties. This approach showed that the natural protein diversity among *L. plantarum *strains cultured in standard conditions can reflect their ability to tolerate bile. The results provided an overview of proteomic patterns related to bile tolerance, and showed a clear effect of bile salts on the level of expression of certain proteins within these patterns. Particularly, 13 out of the 15 proteins of interest were shown to be directly involved in the bile tolerance of *L. plantarum*, six of which could be part of specific bile adaptation pathways, including protection against oxidative stress (GshR1 and GshR4), maintenance of cell envelope integrity (Cfa2), and active removal of bile-related stress factors (Bsh1, OpuA, and AtpH). Also, analysis of changes in protein expression gave insight into the way the different strains use these pathways for their survival, suggesting complex, strain-specific and probably conflicting molecular mechanisms in the cell's adaptation strategy to bile.

Finally, this study showed that comparative proteomic analysis can help understand the differential bacterial properties of LAB. In the field of probiotic studies, characteristic proteomic profiles can be identified for individual properties which may serve as bacterial biomarkers for the preliminary selection of strains with the best probiotic potential. This would certainly increase the chances of success of clinical trials through a more focused approach.

## Methods

### Strain characterization and standard culture conditions

*Lactobacillus *strains used in this study were identified at the species level by *recA *PCR (data not shown) [51]. All cultures were maintained as frozen stocks held at -80°C in Cryobank cryogenic beads (Bio-Rad, Hercules, CA, USA). For experimental use, strains were cultured anaerobically (Anaerocult A system, Merck, Darmstadt, Germany) at 37°C in Man-Rogosa-Sharpe broth (Biokar, Beauvais, France) supplemented with 0.05% (w/v) L-cysteine hydrochloride monohydrate (MRSC; Merck) to early stationary phase, using three successive subcultures (1% v/v inoculation; 12-15 h).

### Bile salt tolerance

Tolerance to bile was assessed by investigating the ability of strains to grow in the presence of different concentrations of bovine bile (Oxgall, Sigma-Aldrich, St Louis, MO, USA), as previously described [52]. Fresh cultures were inoculated (0.1%, v/v) into MRSC broth containing 0.5%, 1.0%, 1.8%, and 3.6% (w/v) Oxgall and incubated anaerobically at 37°C. Bacterial growth was monitored in honeycomb plates (Oy Growth Curves AB, Helsinki, Finland) by measuring the optical density at 600 nm (OD_600_) every 30 min for 48 h using an automated turbidimetric system (Bioscreen C MBR, Oy Growth Curves AB). Three independent experiments were carried out and each assay was performed in triplicate. Comparison of cultures was based on their growth rates in each broth, expressed as a percentage of that of the control which was assigned a value of 100% [52]. Using Statgraphics plus 5.1 software (Manugistics, Rockville, MD, USA), data were subjected to two-way ANOVA with strain and bile concentration as variables. Multiple comparison test using least significant difference procedure was carried out to compare means for which the ANOVA test indicated significant mean differences (p < 0.05).

### Whole cell protein extraction

The following experiments (including 2-DE) were performed for bacterial cells cultured in two different broths (MRSC and MRSC supplemented with 3.6% Oxgall). Early stationary phase cells from a 10-mL broth culture were harvested and washed three times with phosphate-buffered saline (PBS). Cell pellets were resuspended in 2 mL of PBS and cryobeads of these suspensions were prepared in liquid nitrogen. The bacterial beads were ground in liquid nitrogen using a cryogenic grinder (6870 Freezer/Mill, Spex CertiPrep, Stanmore, UK) with three steps of 3 min at a rate of 24 impacts/s. After sample centrifugation (5000 g for 5 min, 4°C), supernatants were filtered through a 0.45-μm pore size filter (Chromafil PET; Macherey-Nagel, Düren, Germany). Protein purification was carried out with Trizol reagent (Euromedex, Souffelweyersheim, France) as previously described [12]. Protein concentrations were determined using Bradford protein assay (Bio-Rad) according to the manufacturer's instructions.

### 2-DE

Protein extracts (150 μg) were loaded onto 17-cm strips with a pH range of 4 to 7 (Bio-Rad), focused for 60,000 V.h, and then separated on a 12% SDS-polyacrylamide gel as reported previously [12]. The gels were stained with Bio-Safe Coomassie (Bio-Rad) and scanned on a GS-800 Calibrated Densitometer (Bio-Rad).

### Image analysis

Image analysis of the 2-DE gels was performed using the PD Quest 8.0.1 software (Bio-Rad). Three gels were produced from independent cultures of each strain and only spots that were present on the three gels were selected for inter-strain comparison. Spot intensities were normalized to the sum of intensities of all valid spots in one gel. For analysis of changes in protein expression during bile salt exposure, a protein was considered to be under- or overproduced when changes in normalized spot intensities were of least 1.5-fold at a significance level of p < 0.05 (Student's t test for paired samples), as previously described [14]. Regarding proteome comparison between strains, proteins were considered differentially produced when spot intensities passed the threshold of a twofold difference (one-way ANOVA, p-value < 0.05), as described previously [12].

### LC-MS analysis

Spots of interest were subjected to tryptic in-gel digestion and analyzed by chip-liquid chromatography-quadrupole time of flight (chip-LC-QTOF) using an Agilent G6510A QTOF mass spectrometer equipped with an Agilent 1200 Nano LC system and an Agilent HPLC Chip Cube, G4240A (Agilent Technologies, Santa Clara, CA, USA), as described previously [12].

Briefly, one microliter of sample was injected using an injection loop of 8 μL, a loading flow rate of 3 μL/min for 4 min and a solvent made of ultra-pure water and acetonitrile (HPLC-S gradient grade, Biosolve, Valkenswaard, The Netherlands) (97/3 v/v) with 0.1% formic acid (98-100%, Merck). For the analytical elution, a 24 min gradient from 3 to 60% of acetonitrile in ultra-pure water with 0.1% formic acid was applied at a flow rate of 300 nL/min. ESI in positive mode with 1850 capillary voltage was used. The data were collected in centroid mode using extended dynamic range at mass range of m/z 200-2000 both in MS1 and MS/MS and using two method with different scanning speed: one slow with a scan rate of 1 spectra/s for both MS1 and MS/MS, and one fast scan rate of 0.25 spectra/s for both MS1 and MS/MS. For data acquisition and data export, MassHunter version B.02.0.197.0 (Agilent Technologies) was used.

### Protein identification

After data acquisition, files were uploaded to the in-house installed version of Phenyx (Geneva Bioinformatics, Geneva, Switzerland) for searching the NCBInr (r. 20090608) database with the following criteria: taxonomy: bacteria; scoring model: ESI-QTOF; parent charge: +2, +3 (trust = medium); single round; methionine oxidation, cysteine carboxyamidomethylation (cysteine treated with iodoacetamide), and phosphorylation as partial modifications; trypsin as digestion enzyme; allowance of two missed cleavages; cleavage mode: normal; parent ion tolerance: 0.6 Da; peptide thresholds: length ≥6, score threshold ≥5.0, identification significance p-value ≤ 1.0E-4, accession number score threshold 6.0, coverage threshold ≥0.2, identified ion series: b; b++;y; y++; allowance of conflict resolution. A publicly available MS/MS search algorithm (Open Mass Spectrometry Search Algorithm, OMSSA, [53]) was used with the same search criteria as described above to confirm protein identities and limit the risk of false positives. On the basis of consensus scoring, only proteins recognized by both database search algorithms at a false positive rate of 5% were considered to be correctly identified [54].

## Authors' contributions

EH carried out strain characterization, bile tolerance assays, as well as proteomic experiments, and drafted the manuscript. PH performed LC-MS analysis, participated in the protein identification, and helped write the manuscript. EI helped perform bile tolerance and proteomic experiments, data analysis and interpretation. FB participated in strain characterization and in revision of the manuscript. EH, EM, DAW, and SE conceived and designed the study. SE helped write the manuscript and revised it. All authors read and approved its final version.

## Supplementary Material

Additional file 1 **Identification of differentially expressed protein spots among *L. plantarum *LC 56, LC 804 and 299 V in standard growth conditions**. The table lists proteins with at least a twofold difference of expression (p-value < 0.05) between the three strains cultured in MRSC. Identification was achieved following excision of differentially expressed spots between gels, tryptic digestion of the corresponding proteins, analysis of the peptide solutions obtained with LC-MS, and proteomic database search. Scores result from proteomic database search using Phenyx. Click here for file

## References

[B1] Turnbaugh PJ Ley RE Hamady M Fraser-Liggett CM Knight R Gordon JI The human microbiome project Nature 2007 449 804 810 10.1038/nature06244 17943116 PMC3709439

[B2] Bäckhed F Ley RE Sonnenburg JL Peterson DA Gordon JI Host microbial mutualism in the human intestine Science 2005 307 1915 1920 15790844 10.1126/science.1104816

[B3] Swidsinski A Loening-Baucke V Vaneechoutte M Doerffel Y Active Crohn's disease and ulcerative colitis can be specifically diagnosed and monitored based on the biostructure of the fecal flora Inflamm Bowel Dis 2008 14 147 161 10.1002/ibd.20330 18050295

[B4] FAO/WHO Guidelines for the evaluation of probiotics in food 2002 London

[B5] Preidis GA Versalovic J Targeting the human microbiome with antibiotics, probiotics, and prebiotics: gastroenterology enters the metagenomics era Gastroenterology 2009 136 2015 2031 10.1053/j.gastro.2009.01.072 19462507 PMC4108289

[B6] Reuter G The *Lactobacillus *and *Bifidobacterium *microflora of the human intestine: composition and succession Curr Issues Intest Microbiol 2001 2 43 53 11721280

[B7] Bernardeau M Guguen M Vernoux JP Beneficial lactobacilli in food and feed: long-term use, biodiversity and proposals for specific and realistic safety assessments FEMS Microbiol Rev 2006 30 487 513 10.1111/j.1574-6976.2006.00020.x 16774584

[B8] Weichselbaum E Probiotics and health: a review of the evidence Nutr Bull 2009 34 340 373 10.1111/j.1467-3010.2009.01782.x

[B9] Senok AC Ismaeel AY Botta GA Probiotics: facts and myths Clin Microbiol Infect 2005 11 958 966 10.1111/j.1469-0691.2005.01228.x 16307549

[B10] Oelschlaeger TA Mechanisms of probiotic actions - a review Int J Med Microbiol 2010 300 57 62 10.1016/j.ijmm.2009.08.005 19783474

[B11] Grossklaus R Codex recommendations on the scientific basis of health claims Eur J Nutr 2009 48 Suppl 1 15 22 10.1007/s00394-009-0077-z 19937439

[B12] Izquierdo E Horvatovich P Marchioni E Aoude-Werner D Sanz Y Ennahar S 2-DE and MS analysis of key proteins in the adhesion of *Lactobacillus plantarum*, a first step toward early selection of probiotics based on bacterial biomarkers Electrophoresis 2009 30 949 956 10.1002/elps.200800399 19309013

[B13] Sanchez B Champomier-Verges MC Anglade P Baraige F Reyes-Gavilan CGD Margolles A Zagorec M Proteomic analysis of global changes in protein expression during bile salt exposure of *Bifidobacterium longum *NCIMB 8809 J Bacteriol 2005 187 5799 5808 10.1128/JB.187.16.5799-5808.2005 16077128 PMC1196055

[B14] Sanchez B Champomier-Verges MC Stuer-Lauridsen B Ruas-Madiedo P Anglade P Baraige F Reyes-Gavilan CGD Johansen E Zagorec M Margolles A Adaptation and response of *Bifidobacterium animalis *subsp *lactis *to bile: a proteomic and physiological approach Appl Environ Microbiol 2007 73 6757 6767 10.1128/AEM.00637-07 17827318 PMC2074956

[B15] Lee K Lee HG Choi YJ Proteomic analysis of the effect of bile salts on the intestinal and probiotic bacterium *Lactobacillus reuteri* J Biotechnol 2008 137 14 19 10.1016/j.jbiotec.2008.07.1788 18680767

[B16] Leverrier P Dimova D Pichereau V Auffray Y Boyaval P Jan GL Susceptibility and adaptive response to bile salts in *Propionibacterium freudenreichii*: physiological and proteomic analysis Appl Environ Microbiol 2003 69 3809 3818 10.1128/AEM.69.7.3809-3818.2003 12839748 PMC165135

[B17] Sanchez B Champomier-Verges MC Collado MD Anglade P Baraige F Sanz Y Reyes-Gavilan CGD Margolles A Zagorec M Low-pH adaptation and the acid tolerance response of *Bifidobactetium longum *biotype *longum* Appl Environ Microbiol 2007 73 6450 6459 10.1128/AEM.00886-07 17720838 PMC2075061

[B18] Lee K Lee HG Pi K Choi YJ Effect of low pH on protein expression by the probiotic bacterium *Lactobacillus reuteri* Proteomics 2008 8 1624 1630 10.1002/pmic.200700663 18351691

[B19] Lorca GL de Valdez GF Ljungh A Characterization of the proteinsynthesis dependent adaptive acid tolerance response in *Lactobacillus acidophilus* J Mol Microbiol Biotechnol 2002 4 525 532 12432952

[B20] Yang F Wang JJ Li XJ Ying TY Qiao SY Li D Wu G 2-DE and MS analysis of interactions between *Lactobacillus fermentum *I5007 and intestinal epithelial cells Electrophoresis 2007 28 4330 4339 10.1002/elps.200700166 18004711

[B21] Beck HC Madsen SM Glenting J Petersen J Israelsen H Norrelykke MR Antonsson M Hansen AM Proteomic analysis of cell surface-associated proteins from probiotic *Lactobacillus plantarum* FEMS Microbiol Lett 2009 297 61 66 10.1111/j.1574-6968.2009.01662.x 19527296

[B22] Enroth H Akerlund T Sillen A Engstrand L Clustering of clinical strains of *Helicobacter pylori *analyzed by two-dimensional gel electrophoresis Clin Diagn Lab Immunol 2000 7 301 306 10702510 10.1128/cdli.7.2.301-306.2000 PMC95866

[B23] Betts JC Dodson P Quan S Lewis AP Thomas PJ Duncan K McAdam RA Comparison of the proteome of *Mycobacterium tuberculosis *strain H37Rv with clinical isolate CDC 1551 Microbiology 2000 146 3205 3216 11101678 10.1099/00221287-146-12-3205

[B24] Duffes F Jenoe P Boyaval P Use of two-dimensional electrophoresis to study differential protein expression in divercin V41-resistant and wildtype strains of *Listeria monocytogenes* Appl Environ Microbiol 2000 66 4318 4324 10.1128/AEM.66.10.4318-4324.2000 11010876 PMC92302

[B25] Wang XS He X Jiang Z Wang J Chen XN Liu DW Wang F Guo Y Zhao J Liu F Huang L Yuan J Proteomic analysis of the *Enterococcus faecalis *V583 strain and clinical isolate V309 under vancomycin treatment J Proteome Res 2010 9 1772 1785 10.1021/pr901216e 20128627

[B26] Aires J Anglade P Baraige F Zagorec M Champomier-Verges MC Butel MJ Proteomic comparison of the cytosolic proteins of three *Bifidobacterium longum *human isolates and *B. longum *NCC2705 BMC Microbiol 2010 10 29 10.1186/1471-2180-10-29 20113481 PMC2824696

[B27] Begley M Gahan CGM Hill C The interaction between bacteria and bile FEMS Microbiol Rev 2005 29 625 651 10.1016/j.femsre.2004.09.003 16102595

[B28] Lebeer S Vanderleyden J De Keersmaecker SCJ Genes and molecules of lactobacilli supporting probiotic action Microbiol Mol Biol Rev 2008 72 728 764 10.1128/MMBR.00017-08 19052326 PMC2593565

[B29] de Vries MC Vaughan EE Kleerebezem M de Vos WM *Lactobacillus plantarum*- survival, functional and potential probiotic properties in the human intestinal tract Int Dairy J 2006 16 1018 1028 10.1016/j.idairyj.2005.09.003

[B30] Molenaar D Bringel F Schuren FH de Vos WM Siezen RJ Kleerebezem M Exploring *Lactobacillus plantarum *genome diversity by using microarrays J Bacteriol 2005 187 6119 6127 10.1128/JB.187.17.6119-6127.2005 16109953 PMC1196139

[B31] Kubota K Kosaka T Ichikawa K Combination of two-dimensional electrophoresis and shotgun peptide sequencing in comparative proteomics J Chromatogr B Analyt Technol Biomed Life Sci 2005 815 3 9 10.1016/j.jchromb.2004.10.030 15652794

[B32] FSA An evaluation of probiotic effects in the human gut: microbial aspects 2004 London

[B33] Gilliland SE Staley TE Bush LJ Importance of bile tolerance of *Lactobacillus acidophilus *used as a dietary adjunct J Dairy Sci 1984 67 3045 3051 10.3168/jds.S0022-0302(84)81670-7 6442304

[B34] Usman Hosono A Bile tolerance, taurocholate deconjugation, and binding of cholesterol by *Lactobacillus gasseri *strains J Dairy Sci 1999 82 243 248 10.3168/jds.S0022-0302(99)75229-X 10068945

[B35] Rallu F Gruss A Ehrlich SD Maguin E Acid- and multistress-resistant mutants of *Lactococcus lactis*: identification of intracellular stress signals Mol Microbiol 2000 35 517 528 10.1046/j.1365-2958.2000.01711.x 10672175

[B36] Burns P Sanchez B Vinderola G Ruas-Madiedo P Ruiz L Margolles A Reinheimer J Reyes-Gavilán CGD Inside the adaptation process of *Lactobacillus delbrueckii *subsp. *lactis *to bile Int J Food Microbiol 2010 142 132 141 10.1016/j.ijfoodmicro.2010.06.013 20621375

[B37] Whitehead K Versalovic J Roos S Britton RA Genomic and genetic characterization of the bile stress response of probiotic *Lactobacillus reuteri *ATCC 55730 Appl Environ Microbiol 2008 74 1812 1819 10.1128/AEM.02259-07 18245259 PMC2268311

[B38] Pfeiler EA Azcarate-Peril MA Klaenhammer TR Characterization of a novel bile-inducible operon encoding a two-component regulatory system in *Lactobacillus acidophilus* J Bacteriol 2007 189 4624 4634 10.1128/JB.00337-07 17449631 PMC1913432

[B39] Chiancone E Ceci P The multifaceted capacity of Dps proteins to combat bacterial stress conditions: detoxification of iron and hydrogen peroxide and DNA binding Biochim Biophys Acta 2010 1800 798 805 20138126 10.1016/j.bbagen.2010.01.013

[B40] Vila-Sanjurjo A Schuwirth BS Hau CW Cate JHD Structural basis for the control of translation initiation during stress Nat Struct Mol Biol 2004 11 1054 1059 10.1038/nsmb850 15502846

[B41] Carmel-Harel O Storz G Roles of the glutathione- and thioredoxindependent reduction systems in the *Escherichia coli *and *Saccharomyces cerevisiae *responses to oxidative stress Annu Rev Microbiol 2000 54 439 461 10.1146/annurev.micro.54.1.439 11018134

[B42] Shabala L Ross T Cyclopropane fatty acids improve *Escherichia coli *survival in acidified minimal media by reducing membrane permeability to H+ and enhanced ability to extrude H+ Res Microbiol 2008 159 458 461 10.1016/j.resmic.2008.04.011 18562182

[B43] Klaenhammer TR Barrangou R Buck BL Azcarate-Peril MA Altermann E Genomic features of lactic acid bacteria effecting bioprocessing and health FEMS Microbiol Rev 2005 29 393 409 10.1016/j.fmrre.2005.04.007 15964092

[B44] Sanchez B Reyes-Gavilan CGD Margolles A The F1F0-ATPase of *Bifidobacterium animalis *is involved in bile tolerance Environ Microbiol 2006 8 1825 1833 10.1111/j.1462-2920.2006.01067.x 16958763

[B45] Bron PA Molenaar D Vos WM Kleerebezem M DNA micro-array-based identification of bile-responsive genes in *Lactobacillus plantarum* J Appl Microbiol 2006 100 728 738 10.1111/j.1365-2672.2006.02891.x 16553727

[B46] Leverrier P Vissers JPC Rouault A Boyaval P Jan G Mass spectrometry proteomic analysis of stress adaptation reveals both common and distinct response pathways in *Propionibacterium freudenreichii* Arch Microbiol 2004 181 215 230 10.1007/s00203-003-0646-0 14730419

[B47] Poolman B Glaasker E Regulation of compatible solute accumulation in bacteria Mol Microbiol 1998 29 397 407 10.1046/j.1365-2958.1998.00875.x 9720860

[B48] Sleator RD Wemekamp-Kamphuis HH Gahan CGM Abee T Hill C A PrfA-regulated bile exclusion system (BilE) is a novel virulence factor in *Listeria monocytogenes* Mol Microbiol 2005 55 1183 1195 10.1111/j.1365-2958.2004.04454.x 15686563

[B49] Lambert JM Bongers RS de Vos WM Kleerebezem M Functional analysis of four bile salt hydrolase and penicillin acylase family members in *Lactobacillus plantarum *WCFS1 Appl Environ Microbiol 2008 74 4719 4726 10.1128/AEM.00137-08 18539794 PMC2519332

[B50] Fang F Li Y Bumann M Raftis EJ Casey PG Cooney JC Walsh MA O'Toole PW Allelic variation of bile salt hydrolase genes in *Lactobacillus salivarius *does not determine bile resistance levels J Bacteriol 2009 191 5743 5757 10.1128/JB.00506-09 19592587 PMC2737978

[B51] Bringel F Castioni A Olukoya DK Felis GE Torriani S Dellaglio F *Lactobacillus plantarum *subsp *argentoratensis *subsp *nov*., isolated from vegetable matrices Int J Syst Evol Microbiol 2005 55 1629 1634 10.1099/ijs.0.63333-0 16014493

[B52] Izquierdo E Medina M Ennahar S Marchioni E Sanz Y Resistance to simulated gastrointestinal conditions and adhesion to mucus as probiotic criteria for *Bifidobacterium longum *strains Curr Microbiol 2008 56 613 618 10.1007/s00284-008-9135-7 18330633

[B53] Geer LY Markey SP Kowalak JA Wagner L Xu M Maynard DM Yang X Shi W Bryant SH Open mass spectrometry search algorithm J Proteome Res 2004 3 958 964 10.1021/pr0499491 15473683

[B54] Kapp EA Schutz F Connolly LM Chakel JA Meza JE Miller CA Fenyo D Eng JK Adkins JN Omenn GS Simpson RJ An evaluation, comparison, and accurate benchmarking of several publicly available MS/MS search algorithms: sensitivity and specificity analysis Proteomics 2005 5 3475 3490 10.1002/pmic.200500126 16047398

[B55] Matuszewska E Kwiatkowska J Kuczynska-Wisnik D Laskowska E *Escherichia coli *heat-shock proteins IbpA/B are involved in resistance to oxidative stress induced by copper Microbiology 2008 154 1739 1747 10.1099/mic.0.2007/014696-0 18524928

[B56] Rajagopal S Sudarsan N Nickerson KW Sodium dodecyl sulfate hypersensitivity of *clpP *and *clpB *mutants of *Escherichia coli* Appl Environ Microbiol 2002 68 4117 4121 10.1128/AEM.68.8.4117-4121.2002 12147516 PMC124035

[B57] Jansch A Korakli M Vogel RF Ganzle MG Glutathione reductase from *Lactobacillus sanfranciscensis *DSM20451(T): contribution to oxygen tolerance and thiol exchange reactions in wheat sourdoughs Appl Environ Microbiol 2007 73 4469 4476 10.1128/AEM.02322-06 17496130 PMC1932818

[B58] Greenberg JT Monach P Chou JH Josephy PD Demple B Positive control of a global antioxidant defense regulon activated by superoxidegenerating agents in *Escherichia coli* Proc Natl Acad Sci USA 1990 87 6181 6185 10.1073/pnas.87.16.6181 1696718 PMC54496

[B59] Biemans-Oldehinkel E Mahmood NABN Poolman B A sensor for intracellular ionic strength Proc Natl Acad Sci USA 2006 103 10624 10629 10.1073/pnas.0603871103 16815971 PMC1502282

[B60] Martinez A Kolter R Protection of DNA during oxidative stress by the nonspecific DNA-binding protein Dps J Bacteriol 1997 179 5188 5194 9260963 10.1128/jb.179.16.5188-5194.1997 PMC179379

[B61] Han XL Dorsey-Oresto A Malik M Wang JY Drlica K Zhao XL Lu T *Escherichia coli *genes that reduce the lethal effects of stress BMC Microbiol 2010 10 35 10.1186/1471-2180-10-35 20128927 PMC2824699

